# Viral but Not Verified: Analyzing Accuracy and Engagement in TikTok Discussions of IUDs

**DOI:** 10.3390/healthcare14111495

**Published:** 2026-05-28

**Authors:** Emily Ahner, Iren Gharibyan, Ann Beck, Samrawit Misiker, Christine Lee, Paige Hewitt, Vidhani Goel, Dale M. Netski, Kavita Batra, Leanne Free, Nadia Gomez

**Affiliations:** 1 Kirk Kerkorian School of Medicine at UNLV, University of Nevada, Las Vegas, NV 89106, USAbecka1@unlv.nevada.edu (A.B.);; 2 Office of Research, Kirk Kerkorian School of Medicine at UNLV, University of Nevada, Las Vegas, NV 89102, USA; 3 School of Public Health, University of Nevada, Las Vegas, NV 89119, USA; 4 Department of Medical Education, Kirk Kerkorian School of Medicine at UNLV, University of Nevada, Las Vegas, NV 89106, USA; 5 Office of Faculty Affairs, Kirk Kerkorian School of Medicine at UNLV, University of Nevada, Las Vegas, NV 89102, USA; 6 Department of Gynecologic Surgery & Obstetrics, Kirk Kerkorian School of Medicine at UNLV, University of Nevada, Las Vegas, NV 89102, USAnadia.gomez@unlv.edu (N.G.)

**Keywords:** intrauterine devices, TikTok, medical misinformation, reproductive health, social media, content analysis, information quality

## Abstract

**Background:** TikTok has emerged as a major source of health information, including content related to intrauterine devices (IUDs). However, the accuracy, quality, and engagement patterns of IUD-related content on this platform remain insufficiently characterized. This study evaluated the informational quality, thematic focus, misinformation prevalence, and engagement metrics of widely viewed IUD-related TikTok videos. **Methods:** A descriptive cross-sectional content analysis was conducted of TikTok videos retrieved using 12 IUD-related search terms. Engagement metrics, creator characteristics, and content features were extracted, including educational, testimonial, and advice-seeking videos. Advice-seeking videos were included to capture user-generated concerns and inquiries that may influence engagement with health-related content on social media platforms. Informational reliability and quality were assessed using the Modified DISCERN instrument and the Global Quality Scale (GQS). Differences across groups were examined using *t*-tests, ANOVA, and chi-square tests. **Results:** A total of 458 videos were included. Nearly half were testimonial or advice-seeking (47.8%), while 38.9% were educational. Most content was produced by non-healthcare creators (76.4%). Engagement metrics did not differ significantly across video type, source, or creator qualification (all *p* > 0.05). Frequently discussed topics included adverse effects (36.5%), insertion experiences (31.2%), and device removal or discontinuation (19.9%). Overall informational quality was low, with mean GQS and Modified DISCERN scores of 2.0 and 1.8, respectively. Physician-created content demonstrated significantly higher quality and reliability scores (both *p* < 0.001). **Conclusions:** Widely viewed IUD-related TikTok content demonstrates high engagement but generally low informational quality.

## 1. Introduction

The rise of social media as a primary source of health information has coincided with a troubling increase in medical misinformation, resulting in significant consequences for public health [1]. A recent, notable example of these consequences is the resurgence of measles outbreaks driven by vaccine hesitancy, a phenomenon fueled in part by misinformation circulating on social media platforms [2,3]. Additionally, apps such as TikTok have become popular destinations for individuals seeking health advice; however, evidence suggests that inaccurate posts often generate more user engagement than those sharing accurate information [4,5].

This trend is particularly concerning in the field of sexual and reproductive health, where misinformation can lead to lasting and sometimes irreversible effects [6]. As access to comprehensive sex education continues to decline in the United States (U.S.), many individuals turn to platforms like TikTok to fill these educational gaps [7]. Recent studies show that posts on sexual and reproductive health by non-experts often gain more traction than those created by healthcare professionals, further amplifying inaccuracies [4,8].

Contraceptive methods, especially intrauterine devices (IUDs), are a common topic of interest on TikTok [9,10]. Intrauterine devices (IUDs) were selected as the focus of this study because they represent one of the most effective forms of long-acting reversible contraception, with well-established safety and efficacy profiles and typical-use failure rates of less than 1% [11,12]. Despite strong clinical evidence supporting both hormonal and copper IUDs, these methods are frequently portrayed on social media as harmful, with numerous systemic side effects attributed to their use [13]. This disconnect between evidence-based guidance and public perception makes IUDs particularly vulnerable to misinformation. Intrauterine devices (IUDs) have both a high efficacy and safety profile, but they remain the subject of widespread debate. Both hormonal and copper IUDs are commonly portrayed as harmful, with many systemic side effects attributed to their use [13]. The emphasis TikTok places on personal storytelling in place of clinical evidence compounds this issue, as anecdotal posts are more likely to go viral and, in some cases, may oversimplify or distort scientific facts [14,15,16,17]. Given the platform’s widespread use among reproductive-age individuals, inaccurate or low-quality information has the potential to meaningfully influence contraceptive decision-making. Furthermore, IUD-related TikTok content is frequently of low educational quality and lacks standardized assessment, leading to significant variability in the information presented [18,19]. Despite the growing influence of TikTok as a source of health information, there remains a need for systematic evaluation of IUD-specific content, which this study aims to address.

While previous research has explored health misinformation on social media more broadly, few studies have specifically examined public attitudes toward IUDs, and existing work is largely limited to qualitative analyses or platform-specific studies that emphasize user experiences and narratives rather than systematically evaluating informational quality, engagement patterns, or misinformation [20,21]. Existing literature often focuses on other contraceptive methods or broader health misinformation topics, with relatively limited attention to IUD-specific content on social media platforms [4,8,20]. The health-related content on social media is frequently characterized by a high prevalence of misinformation, a dominance of non-healthcare creators, and a strong reliance on anecdotal narratives that drive user engagement [1,4,8]. Prior studies have also shown that contraceptive-related discussions on platforms such as TikTok are often shaped by personal experiences and may lack evidence-based information [4,8,20]. However, important gaps remain. Few studies have focused specifically on IUD-related content, and even fewer have simultaneously evaluated informational quality, engagement patterns, and misinformation within a unified framework. Additionally, most existing research does not differentiate between physicians, non-physician healthcare providers, and non-healthcare creators, limiting a more nuanced understanding of content quality across creator types. Addressing these gaps is essential to better understand how reproductive health information is presented and consumed on rapidly evolving social media platforms.

To address these gaps, this study aimed to evaluate the reliability, quality, thematic content, and prevalence of misinformation in widely viewed TikTok videos related to intrauterine devices (IUDs). In addition, we aimed to examine differences in engagement patterns across creator types, including healthcare professionals and non-healthcare creators, and to assess whether engagement is associated with informational reliability and creator background. A structured summary of the current evidence and identified research gaps is presented in Table 1.

## 2. Methods

### 2.1. Search Strategy and Data Collection

This study was deemed exempt by the University of Nevada, Las Vegas Institutional Review Board (UNLV-2022-169; approved 30 March 2022), as it involved the analysis of publicly available social media content. The TikTok video search was conducted on 12 February 2024, using a standardized protocol to ensure consistency and reproducibility of results. To minimize the influence of prior user behavior on TikTok’s recommendation algorithm, a new email address and TikTok account were created specifically for data collection. All reviewers used this account exclusively for the purposes of this study. Searches were conducted on TikTok’s web platform (https://www.tiktok.com) using the following terms: “IUD,” “intrauterine device,” “hormonal IUD,” “Mirena,” “Liletta,” “Kyleena,” “Skyla IUD,” “levonorgestrel IUD,” “LNG IUD,” “non-hormonal IUD,” “copper IUD,” and “Paragard.” Each term was entered individually into TikTok’s search bar without the use of Boolean operators, as the platform does not support advanced search syntax. For each search term, the first 100 uniform resource locators (URLs) returned by TikTok’s default algorithm were collected on the same day and compiled into a master database to capture content with the greatest visibility and user reach.

### 2.2. Inclusion and Exclusion Criteria

Videos were eligible for inclusion if they were uploaded to TikTok from a public account, presented in English, and contained content relevant to hormonal or non-hormonal IUDs. Videos were excluded if they were not in English or if the content was unrelated to IUDs. Two authors independently screened all videos for eligibility, and discrepancies were resolved by a third author. All eligible video URLs were compiled into a master database. Manual verification was performed to identify and remove duplicate entries resulting from formatting inconsistencies. Because the initial pool of eligible videos exceeded the scope for detailed content analysis, the dataset was restricted to the top 500 most-viewed videos across all search terms to prioritize content with the greatest potential audience reach. Videos that were deleted or became unavailable prior to analysis were excluded, yielding a final analytic sample of 458 videos.

### 2.3. Data Extraction and Coding

Data were extracted using a structured spreadsheet designed to capture key characteristics of each video. Engagement metrics included total views, likes, comments, saves, shares, and follower counts. Derived engagement measures such as views per day, views per like, and likes per day were calculated by dividing total views or likes by the number of days between the video upload date and the date of analysis. Additional variables included whether the video was sponsored and whether the creator’s account was verified by TikTok, which indicates that the account belongs to the person or brand it represents [22,23]. Engagement metrics were limited to quantitative measures, and qualitative analysis of user comments was not performed.

Creator- and content-level characteristics were also recorded. The content category reflected the primary theme of each video and was categorized as educational/informational, testimonial, seeking advice, advertisement, humor/entertainment, political, or other. Sources referred to the affiliation associated with the account (e.g., physician, physician group, pharmacist, non-physician healthcare provider (non-HCP), patient, educational platform, comedy platform, medical advertisement, for-profit company, other organization, another individual user, student, or other). Creator qualifications reflected the individual creator’s reported professional or experiential background (educator, healthcare student, non-physician healthcare provider or scientist, none, other, or physician) and were later grouped for purposes of data analysis into physician, non-physician healthcare provider, and other. Content categories and coding variables were developed a priori based on existing literature and refined through pilot testing to ensure consistency in interpretation across reviewers. Advice-seeking videos were included to capture user-generated inquiries and concerns related to IUDs, as these posts contribute to the broader informational environment and may influence how audiences interpret and engage with health-related content on social media platforms.

Additional characteristics included the perceived sex and race of the creator, mode of information delivery (individual(s) in the video, external voice, no speaker, text, or other), type of IUD discussed (e.g., hormonal, non-hormonal), and the specific IUD brand name when mentioned. Creator sex and race were recorded based on reviewer-perceived characteristics from publicly available content and were included for descriptive purposes only; these variables were not used as primary analytical predictors in inferential analyses. Content-specific information was captured using standardized dropdown categories, including topics discussed, positive and negative effects of continuation, positive and negative effects of discontinuation, the creator’s overall attitude toward IUDs, and any false claims identified. Prior to data extraction, reviewers completed pilot coding to ensure consistent interpretation of coding definitions and study variables. Formal inter-rater agreement statistics (e.g., kappa coefficients) were not calculated for non-quality variables. These variables were coded using predefined categories following pilot testing to ensure consistency, and discrepancies were resolved through discussion with a third reviewer. All statements identified as false or requiring factual verification were independently reviewed by a physician subject matter expert using current evidence-based clinical guidelines, including American College of Obstetricians and Gynecologists (ACOG) practice bulletins, the Centers for Disease Control and Prevention (CDC) U.S. Selected Practice Recommendations for Contraceptive Use (2024), and U.S. Medical Eligibility Criteria for Contraceptive Use (2024), as well as relevant peer-reviewed literature [22,23]. The use of multiple concordant guideline sources minimized the likelihood of misclassification due to differences in clinical recommendations [11,24,25,26].

Content was not classified as a false claim if it reflected personal experiences, subjective interpretations, or incomplete information without making objectively verifiable incorrect statements. For example, anecdotal reports of adverse effects (e.g., pain, bleeding) were not considered false claims unless they were generalized as universal outcomes or presented with factually incorrect assertions. In contrast, statements such as “IUDs are unsafe for all individuals” or claims attributing unsupported systemic effects were classified as false claims. Borderline or ambiguous cases were reviewed conservatively, with preference given to avoiding overclassification of misinformation.

### 2.4. Quality and Reliability of Information

Each video was independently assessed by two reviewers who were blinded to each other’s scores. Information reliability was evaluated using the Modified DISCERN criteria, which consists of five binary yes/no criteria adapted for short-form social media content: (1) clarity and achievement of aims; (2) use of reliable information sources; (3) balance and lack of bias; (4) provision of additional sources for viewer reference; and (5) acknowledgment of areas of uncertainty. Each criterion was scored as 1 (“yes”) or 0 (“no”), resulting in a total reliability score ranging from 0 (low reliability) to 5 (high reliability) [24]. Overall video quality was assessed using the Global Quality Scale (GQS), a 5-point Likert scale that assesses information flow, completeness, and usefulness of information to viewers. The Global Quality Scale’s ratings ranged from 1 (“poor quality, poor flow, and minimal usefulness”) to 5 (“excellent quality, high completeness, and high usefulness”).

Final Modified DISCERN and GQS scores for each video were calculated by averaging the two reviewers’ ratings. Inter-rater reliability was evaluated prior to averaging reviewer scores. Reviewers were blinded to engagement metrics (e.g., views, likes, comments) during quality scoring to minimize potential bias. When notable differences in interpretation arose, reviewers discussed the coding criteria to ensure consistency, but the final scores were not changed through consensus. This approach is consistent with prior social media content analyses using Modified DISCERN and GQS scoring frameworks [27,28]. The Global Quality Scale (GQS) was selected for this study due to its widespread use in evaluating health-related content on video-based and social media platforms. Compared to other tools such as the Patient Education Materials Assessment Tool (PEMAT), the GQS is particularly suited for short-form content, as it provides a global assessment of information quality, flow, and usefulness without requiring extensive textual material. This makes it appropriate for evaluating TikTok videos, which are typically brief and may not meet the structural requirements of traditional patient education assessment tools.

### 2.5. Statistical Analysis

The dataset was reviewed for completeness and internal consistency prior to analysis. All statistical analyses were conducted using IBM SPSS Statistics (version 28; IBM Corp., Armonk, NY, USA). Descriptive and bivariate statistical analyses were used to examine the characteristics, engagement patterns, and informational quality of TikTok videos related to intrauterine devices (IUDs). Categorical variables were summarized as frequencies and percentages with corresponding 95% confidence intervals. Continuous variables are summarized using medians and interquartile ranges (Q1–Q3), given the skewed distribution of engagement metrics. Mean (standard deviation) values are retained for consistency with parametric statistical analyses. Independent-sample *t*-tests were conducted to compare mean engagement metrics (likes, views, and comments) across video and creator characteristics, including educational versus non-educational content, individual versus organizational creators, and physician versus non-physician creators. These analyses were exploratory and intended to identify differences in engagement patterns across groups. Inter-rater reliability for Modified DISCERN and Global Quality Scale (GQS) scores was assessed using the intraclass correlation coefficient (ICC), based on a two-way random-effects model with absolute agreement. Comparisons of informational quality by creator qualification were conducted using omnibus ANOVA for continuous outcomes (mean Modified DISCERN and GQS scores). However, Levene’s test indicated a violation of the homogeneity of variance assumption (*p* < 0.001). Accordingly, Games–Howell post hoc comparisons were conducted to account for unequal variances and group sizes. Mean differences are reported for interpretability within the context of parametric post hoc comparisons, despite the skewed distribution of engagement data. Given the exploratory nature of this study and the large sample size, mean comparisons were used to facilitate interpretability despite skewed distributions. Engagement metrics (e.g., views, likes, comments) demonstrated substantial right-skewed distributions. Despite this, parametric tests (independent-sample *t*-tests and ANOVA) were used for group comparisons. These methods are well established to be robust to violations of normality, particularly in moderate to large samples, due to the Central Limit Theorem and the approximate normality of sampling distributions of the mean. With the sample size in this study (*n* = 458), parametric approaches are considered appropriate and statistically valid for inference. Furthermore, prior methodological research has demonstrated that parametric tests maintain acceptable Type I error rates and statistical power under non-normal conditions [29,30]. Parametric methods also provide greater interpretability and statistical power compared to non-parametric alternatives. To ensure transparency, both mean (standard deviation) and median (interquartile range) values are reported.

Differences in the presence of false claims between physician and non-physician creators were assessed using chi-square tests. A two-sided *p*-value of <0.05 was considered statistically significant. Comparisons of informational quality by creator qualification were also assessed using one-way analysis of variance (ANOVA). Prior to post hoc testing, Levene’s test for homogeneity of variances was evaluated. Due to significant violations of the homogeneity assumption (Levene’s *p* < 0.001), pairwise post hoc comparisons were conducted using Games–Howell tests, which account for unequal variances and sample sizes. Games–Howell *p*-values, pairwise comparisons, and 95% confidence intervals were reported to identify statistically significant differences between physician, non-physician healthcare provider, and other creator groups.

## 3. Results

During data cleaning, 61 duplicate entries were identified and removed prior to final sample selection. Following this process, a total of 458 videos were included in the final analysis. The characteristics of TikTok videos related to intrauterine devices (IUDs) and their creators are summarized in Table 2. Content was predominantly testimonial or advice-seeking, with a substantial proportion of educational videos, while other content types (e.g., advertisements, humor, political content) were less common. Most videos were produced by individual, non-healthcare creators, with relatively limited representation from healthcare-trained professionals. Verified and sponsored content comprised only a small proportion of the sample. Sponsored content was rare, accounting for only a small proportion of videos (approximately 1%), limiting further subgroup analysis.

Video-level engagement metrics and informational quality scores are summarized in Table 3. Engagement measures demonstrated substantial variability, with distributions indicating that a small subset of videos accounted for disproportionately high levels of interaction. In contrast, informational quality was consistently low across videos, with limited variation in both Modified DISCERN and Global Quality Scale (GQS) scores.

The thematic content of IUD-related TikTok videos is presented in Table 4. Content was primarily centered on experiential narratives, with topics related to adverse effects, insertion experiences, and device removal dominating the discussion. Instructional and clinically oriented content was relatively uncommon, indicating a limited emphasis on evidence-based guidance within widely viewed videos.

Inter-rater reliability for quality assessment was moderate for Modified DISCERN scores and good for GQS scores, indicating consistent agreement between reviewers (Table 5).

Comparisons of user engagement across video type, source, and creator qualification are presented in Table 6. Engagement metrics did not differ significantly across these categories, suggesting that average interaction levels were comparable regardless of content type or creator background. Although variability in engagement was observed, no consistent pattern indicated that specific creator groups or content types achieved systematically higher engagement.

Differences in informational quality by creator qualification are shown in Table 7. Videos created by physicians and non-physician healthcare providers demonstrated higher reliability and quality scores compared with those produced by other creators, indicating a graded pattern of informational quality across creator groups.

Pairwise comparisons of informational quality scores using the Games–Howell post hoc test are presented in Table 8. These analyses indicated that healthcare-trained creators, particularly physicians, had significantly higher informational quality scores compared with non-healthcare creators, while differences between physicians and non-physician healthcare providers were not statistically significant.

## 4. Discussion

### 4.1. Principal Findings

This study provides a comprehensive analysis of widely viewed TikTok videos related to intrauterine devices (IUDs), examining engagement patterns, creator characteristics, informational quality, and the presence of misinformation. IUD-related discourse on TikTok was dominated by testimonial and advice-seeking content, with educational material representing a smaller proportion of videos. Most content was produced by non-healthcare creators, while physicians and non-physician healthcare providers together represented a minority of content creators.

Overall informational quality was low, as reflected by low mean Modified DISCERN and GQS scores. Inter-rater reliability was moderate to good across scoring instruments. Importantly, informational quality differed by creator qualification. Physician-created content demonstrated the highest average reliability and quality, followed by content produced by non-physician healthcare providers, with substantially lower scores observed among other creators. In contrast, engagement metrics did not differ meaningfully across creator groups, suggesting that differences in informational quality were not associated with differences in average engagement levels.

Thematic analysis revealed that adverse effects, insertion experiences, and device removal or discontinuation were the most frequently discussed topics, suggesting that TikTok discourse surrounding IUDs emphasizes experiential and often challenging aspects of use. Although false claims were numerically less frequent in physician-created content, their prevalence did not differ significantly across creator groups, underscoring the widespread circulation of mixed-quality information across the platform.

### 4.2. Comparison with Current Literature

These findings are consistent with prior research demonstrating that contraceptive-related content on TikTok is largely produced by non-healthcare creators and frequently relies on personal narratives, often at the expense of evidence-based information [3,20]. Similar to previous analyses of IUD-related social media content, adverse effects and procedural concerns were the most commonly discussed topics, reinforcing concerns that online discourse may disproportionately emphasize negative experiences [6,20]. Prior research has suggested that misinformation on video-sharing platforms may be associated with emotionally engaging narratives rather than evidence-based explanations [28]. Similar patterns have been observed in analyses of mental health content on short-form video platforms [28,29,30]. These findings are consistent with our observation that experiential content predominates in widely viewed videos.

Our findings are consistent with prior studies examining IUD-related content on social media platforms. Nguyen and Allen (2018) reported that YouTube videos on IUDs frequently combined educational content with personal narratives, contributing to variability in informational quality. Similarly, Allen et al. (2025) found that TikTok content is heavily shaped by user experiences, particularly narratives emphasizing adverse effects and negative clinical interactions [31]. In alignment with these studies, our analysis demonstrates that IUD-related content is largely driven by experiential narratives and exhibits generally low informational quality. However, our study extends this literature by integrating engagement metrics with quality assessment and by distinguishing between physician, non-physician healthcare provider, and non-healthcare creators, providing a more nuanced understanding of content dynamics in high-reach social media environments [31,32].

This study extends the existing literature by differentiating between physicians, non-physician healthcare providers, and non-healthcare creators, rather than treating healthcare professionals as a single group. While both physician and non-physician healthcare provider content demonstrated higher informational quality than content produced by other creators, physician-created videos consistently achieved the highest reliability scores. These findings suggest a graded pattern of informational quality across creator types, highlighting important distinctions within healthcare-trained contributors that have been underexplored in prior social media research.

### 4.3. Interpretation of Results

One of the most notable findings of this study was that informational reliability differed by creator qualification. Physician-created videos demonstrated the highest reliability scores, followed by content from non-physician healthcare providers, while content produced by other creators consistently showed lower reliability. In contrast to expectations, engagement metrics did not differ significantly across creator categories, suggesting that informational quality was not associated with higher or lower levels of user interaction in this dataset.

The absence of significant differences in false claims and engagement across creator groups contrasts with prior research suggesting that non-healthcare creators may be more likely to disseminate misinformation [4,8]. However, this discrepancy may reflect differences in study design, including the restriction to highly viewed videos and the use of conservative classification criteria. Several factors may explain this discrepancy. First, the restriction to the most-viewed videos may have introduced a selection effect, capturing content that had already achieved high visibility regardless of the creator’s background. Second, the use of a structured and conservative definition of false claims based on clinical guidelines may have resulted in a narrower classification compared to broader definitions of misinformation used in other studies [1]. Finally, differences in topic specificity, platform dynamics, and temporal factors may also contribute to variation across findings. These considerations highlight the importance of interpreting engagement and misinformation patterns within the context of study design and content selection. These findings suggest a potential opportunity to expand the presence of evidence-based reproductive health content on social media platforms. However, given the cross-sectional nature of the data, these observations should not be interpreted as evidence of equivalent algorithmic promotion or causal relationships between content quality and engagement.

Our findings also reinforce established patterns in online reproductive health discourse, including the dominance of anecdotal narratives and the disproportionate emphasis on adverse or challenging experiences [5,6]. The prominence of insertion pain, adverse effects, and device removal within negatively framed content suggests that individuals seeking information on social media are frequently exposed to narratives that may amplify fear and uncertainty. This pattern is particularly consequential for IUDs, which already demonstrate lower baseline acceptability compared with other contraceptive methods [16]. Clinically, the predominance of negative or experiential TikTok content is meaningful, as patients often encounter these narratives prior to clinical consultation, potentially shaping expectations and decision-making. Expanding access to accurate, accessible online information may therefore support more effective counseling and patient trust.

Collectively, these results affirm TikTok’s importance as a platform for reproductive health information while challenging the assumption that unreliable or sensationalized content is inherently amplified. Rather, the limited availability of accurate IUD-related information appears to reflect low participation by healthcare-trained creators, as physicians and non-physician healthcare providers represented a minority of content creators despite producing the highest-quality information. The absence of engagement penalties for healthcare-trained creators highlights an opportunity for clinicians and professional organizations to expand their digital presence and improve the quality of reproductive health information available to users. Understanding the IUD-related social media content, physicians and non-physician healthcare providers have the opportunity to discuss potential adverse effects and procedural concerns, such as insertion or pain, using evidence-based literature [33,34,35,36,37]. This will create more reliable and meaningful social media content.

### 4.4. Mechanisms or Explanations

This study provides insight into the characteristics of widely viewed reproductive health content on social media platforms. The predominance of testimonial and experiential content suggests that highly viewed videos may reflect audience interest in personal narratives and lived experiences. The consistently low informational quality observed in this study indicates that engagement alone does not reflect the reliability of content. Given that most videos were produced by non-healthcare creators, audiences may encounter a wide range of information quality. The comparable engagement observed across creator groups suggests that factors other than informational quality may contribute to user interaction.

### 4.5. Implications of the Findings

These findings have important implications for clinical practice, public health communication, and digital health literacy. This study highlights the growing role of social media as a source of health information outside traditional clinical encounters and underscores the variability in informational quality available to users. In the context of increasing concern regarding health misinformation, understanding how individuals access and interpret online health content is essential for informing communication strategies [4]. The predominance of testimonial and experiential content observed in this study suggests that individuals may be exposed to narratives emphasizing adverse effects, insertion experiences, and device removal prior to clinical consultation.

Clinically, the low overall informational quality of IUD-related content suggests that patients may encounter information that is incomplete or not evidence-based, which may influence their perceptions and questions during clinical interactions. Importantly, these commonly discussed topics reflect patient concerns and may provide clinicians with insight into areas that warrant proactive discussion during contraceptive counseling.

From a public health perspective, the coexistence of high engagement and variable informational quality highlights the need to improve the accessibility and visibility of accurate, evidence-based reproductive health information on social media platforms. The comparable engagement observed across creator groups suggests that increased participation by healthcare-trained professionals may improve the informational environment without necessarily compromising reach.

Variability in access to comprehensive sexual education may contribute to reliance on social media platforms for contraceptive information. Prior research has demonstrated differences in contraceptive knowledge and comfort discussing reproductive health topics, which may influence how individuals seek and interpret information online [33,34,35,36,37,38]. In this context, social media platforms may serve as both an accessible source of information and a potential source of variability in informational quality.

The observation that healthcare-trained creators produced higher-quality content suggests an opportunity to expand evidence-based communication on social media. By addressing commonly discussed topics such as adverse effects, insertion experiences, and device removal, clinicians may provide accurate and accessible information that aligns with user interests. However, these findings should be interpreted cautiously, as the study design does not allow for conclusions regarding causal relationships or platform-specific content promotion mechanisms. Future research should explore strategies to enhance the visibility of reliable content, evaluate the impact of clinician-generated content on user knowledge and decision-making, and examine how health-related information is disseminated across social media networks. Longitudinal studies may also provide insight into how content trends evolve over time.

### 4.6. Strengths and Limitations

Several strengths of this study should be noted. The high sample size of videos offers helpful generalizability of the study to the content that the average person on TikTok encounters. The study analyzed 458 videos, providing a robust dataset across multiple video types, creator demographics, and engagement levels. This large sample enhances representativeness and allows for meaningful subgroup comparisons. The use of consistent data collection for video characteristics, creator attributes, content topics, and attitude added consistency and depth to the analysis. Two independent reviewers coded Modified DISCERN and GQS scores, strengthening the reliability of quality assessments. By integrating quantitative engagement data with qualitative topic analysis (adverse effects, insertion experiences, provider interactions), the study provides a multi-dimensional understanding of how IUD-related information circulates on TikTok.

The study demonstrates multiple limitations that should be addressed. Findings may not be generalizable to other social media platforms, such as Facebook, Instagram, Twitter, Reddit, etc., since TikTok was exclusively the platform from which data were gathered. Because TikTok’s search and recommendation algorithms are dynamic, personalized, and not fully transparent, the order and composition of videos returned for each search term may vary over time and between users, which may limit the reproducibility and generalizability of the exact video sample. Furthermore, user demographics and algorithmic behavior differ across platforms, limiting cross-platform applicability. Another limitation includes the differences in individual activity on the platform. Individual activity on TikTok creates a unique algorithm for each user, decreasing the generalizability of our TikTok search. While Modified DISCERN and GQS assess quality and reliability, they do not provide a complete picture of the nuanced misinformation, sensationalism, or misinterpretation of legitimate side effects, all common themes in reproductive health content. Videos were posted across a wide time span (19–1670 days since upload), but the study design did not examine how content quality or engagement trends change over time, or how major policy shifts may influence content. Additionally, the identification and classification of false claims were conducted by a single physician subject matter expert. While this approach ensured clinical accuracy using evidence-based guidelines, it may introduce some degree of subjectivity. Inter-rater reliability was not assessed for false claim adjudication, which may affect reproducibility. Future studies should incorporate multiple clinical reviewers or a formal reliability assessment to strengthen consistency in misinformation classification. Next, the restriction of our sample to the most-viewed videos may have introduced selection bias by focusing on content that had already achieved high visibility. As a result, these findings may not reflect engagement patterns across the full spectrum of TikTok content, particularly for videos with lower reach. It is possible that clinician-produced content with lower engagement was underrepresented in this sample, limiting our ability to assess differences across creator groups more comprehensively. Additionally, highly viewed content may share characteristics related to presentation style, timing, or audience appeal that transcend creator background, potentially reducing observable differences in engagement. These considerations suggest that our findings are most applicable to widely viewed content and should not be generalized to all TikTok videos. Creator sex and race were coded based on reviewer-perceived characteristics derived from publicly available content, which may introduce misclassification bias and not necessarily reflect individuals’ self-identified identities. This approach may also inadvertently reinforce assumptions or inaccuracies related to identity. These variables were included for descriptive purposes only and were not central to the primary analytical objectives of the study. Findings related to these characteristics should therefore be interpreted with caution. Future research should consider alternative approaches, such as self-reported or platform-verified demographic data, where available, to improve accuracy and reduce bias.

Also, while this study included quantitative engagement metrics such as views, likes, comments, shares, and saves, it did not assess the qualitative nature of user interactions. Specifically, we did not evaluate comment content, including sentiment, misinformation within comments, or the presence of insults, stigma, or profanity. Analysis of such interactions would require a separate coding framework or natural language processing approaches and was beyond the scope of the current study. Future research should examine the qualitative dynamics of user engagement to better understand how audiences interpret and respond to health-related content on social media platforms. While this study provides a comprehensive descriptive analysis of widely viewed IUD-related TikTok content, it did not include multivariable or stratified analyses examining associations between specific content characteristics (e.g., topic, tone, or delivery style) and informational quality or engagement outcomes. Given the size of the dataset, such analyses may have provided additional insight into the factors influencing content performance and quality. However, the exploratory nature of this study, the highly skewed distribution of engagement metrics, and the focus on high-reach content limited the interpretability of more complex modeling approaches. Future research should incorporate multivariable analytical frameworks to better understand how content characteristics influence the quality and dissemination of health information on social media platforms. Lastly, the low prevalence of sponsored content in this sample limited our ability to assess its potential influence on engagement or informational quality. Future studies should further examine the role of sponsored or promotional health content on social media platforms.

This study also raises important ethical considerations related to the analysis of publicly available social media content. Although all videos were obtained from public accounts and did not involve direct interaction with individuals, many posts reflect personal health experiences that were not originally intended for research purposes. Care was taken to analyze content at an aggregate level without identifying individual users in order to preserve privacy and minimize potential harm. Additionally, the interpretation of user-generated content was approached with sensitivity to avoid misrepresentation or stigmatization. These considerations highlight the importance of ethical reflection when conducting research using social media data.

## 5. Conclusions

This analysis demonstrates that IUD-related content on TikTok achieves substantial visibility despite generally low informational quality, with most videos produced by non-healthcare creators. While healthcare-trained creators produced higher-quality and more reliable content, engagement metrics did not differ meaningfully across creator groups. These findings highlight the coexistence of high visibility and variable informational quality in social media-based reproductive health content. However, given the cross-sectional design, these results should be interpreted cautiously and do not imply causal relationships or platform-specific algorithmic effects. Further research is needed to better understand the factors influencing content quality, dissemination, and user engagement on social media platforms.

## Figures and Tables

**Table 1:** Evidence gaps in social media research on contraceptive content.

Prior Study Area	Key Findings	Remaining Gap
Health misinformation on social media [1,4]	High prevalence of misinformation; inaccurate content often gains more engagement	Limited focus on contraception-specific misinformation, particularly IUD-related content
Contraceptive content on TikTok (mostly oral contraceptives) [4,8,20]	Content dominated by non-healthcare professionals; low informational quality; anecdotal narratives frequently go viral	Few analyses focused specifically on IUD-related content; limited evaluation integrating quality, engagement, and misinformation
Reproductive health narratives and personal storytelling [14,15,16,17]	Users rely heavily on anecdotal experiences; misinformation may influence reproductive decision-making	Limited understanding of how IUD-related experiences (e.g., insertion, adverse effects, removal) are framed on social media
Healthcare provider presence in digital health spaces [16,17]	Healthcare professional–generated content is less common and often perceived as less engaging	No studies assessing predictors distinguishing healthcare professionals versus non-healthcare creators in contraception-related content
Quality assessment of health-related social media videos (DISCERN/GQS studies) [18,19]	Social media health videos frequently demonstrate low informational quality	No standardized quality assessment has been applied to IUD-related TikTok videos

**Table 2:** Characteristics of TikTok videos and creators (n = 458).

Variable	Categories	n (%)	95% CI (LCL–UCL)
Characteristics of videos			
Video type	Educational/informational	178 (38.9)	(34.4–43.5)
	Testimonial/seeking advice	219 (47.8)	(43.2–52.5)
	Others—including advertisements, Humor/entertainment and political videos	61 (13.3)	(10.3–16.8)
Video source (as reported by the video creator)	Individual	421 (91.9)	(89.1–94.3)
	Organization	37 (8.1)	(5.8–11.0)
Verified	Yes	62 (13.5)	(10.5–17.1)
	No	396 (86.5)	(83–89.5)
Sponsored	Yes	5 (1.1)	(0.4–2.5)
	No	452 (98.7)	(97.2–99.5)
Mode of delivery in the video	External voice including other	28 (6.1)	(4.1–8.7)
	Individual(s) in the video	281 (61.4)	(56.7–65.9)
	No speaker	29 (6.3)	(4.3–9)
	Text	116 (25.3)	(21.4–29.6)
	Other	4 (0.9)	(0.2–2.2)
Overall Attitude (perceived by reviewer)	Positive	85 (18.6)	(15.1–22.4)
	Neutral	225 (49.1)	(44.5–53.8)
	Negative	136 (29.7)	(25.5–34.1)
Characteristics of the video creators			
Gender	Female	415 (90.6)	(87.6–93.1)
	Males	22 (4.8)	(3–7.2)
	Could not determine	19 (4.1)	(2.5–6.4)
Race	White	298 (65.1)	(60.5–69.4)
	Black	68 (14.8)	(11.7–18.4)
	Asian	32 (7)	(4.8–9.7)
	Others	24 (5.2)	(3.4–7.7)
Qualification	Physician	108 (23.6)	(19.8–27.7)
	Non-physician healthcare providers	65 (14.2)	(11.1–17.7)
	Others	285 (62.2)	(57.6–66.7)

n = frequency, CI = confidence interval, LCL = lower confidence limit, UCL = upper confidence limit. Note: Creator gender and race were based on reviewer-perceived characteristics from publicly available video content and profile information. Creator qualification was determined using self-reported credentials provided by the content creator in the video or account profile.

**Table 3:** Descriptive statistics of social media engagement activities, Modified DISCERN, and GQS scores.

Video Characteristics	Minimum	Maximum	Median (Q1, Q3)	Mean (SD)
Days since upload	19	1670	558 (372.5, 850.0)	646.3 (369.5)
Views (×10^6^)	0.0008	90.2	0.113 (0.038, 0.447)	1.14 (5.10)
Likes (×10^3^)	0.013	2400	3.0 (0.6, 17.5)	60.8 (247.8)
Comments (×10^3^)	0	28.6	0.10 (0.03, 0.36)	0.83 (2.88)
Saves (×10^3^)	0	116.1	0.17 (0.04, 0.88)	2.37 (10.11)
Shares (×10^3^)	0	216.4	0.10 (0.02, 0.74)	3.12 (16.58)
Views/day	1	168,421.1	222.5 (63, 867)	2592.6 (11,202.3)
Views/like	4	2607.7	42.3 (21, 72)	60.9 (131.6)
Likes/day	0	6451.7	5.1 (1, 33)	134.3 (578.2)
Creator account followers (×10^3^)	0.039	10,700	47.1 (4.0, 457.4)	472.3 (976.1)
Creator account total likes (×10^6^)	0.0002	250	1.9 (0.18, 14.5)	13.83 (28.15)
Average GQS	1	4.0	2.0 (2.0, 2.0)	2.0 (0.5)
Average Modified DISCERN score	0.5	4.5	1.5 (1.0, 3.0)	1.8 (0.9)

Engagement metrics demonstrated a substantial right-skewed distribution; therefore, median and interquartile range (Q1–Q3) are presented as the primary descriptive statistics. Mean (standard deviation) values are retained for consistency with parametric statistical analyses. Views are presented in millions (×10^6^), and likes, comments, saves, shares, and follower counts are presented in thousands (×10^3^).

**Table 4:** Topics discussed by video creator.

Topic	n	% of Videos Discussing Each Topic (n = 458)	95% CI (LCL–UCL)	% of All Topics Mentions (n = 502)	95% CI (LCL–UCL)
Device removal/discontinuation	91	19.9%	(16.3–23.8)	18.1%	(14.9–21.8)
Device insertion	143	31.2%	(27.0–35.7)	28.5%	(24.6–32.7)
Adverse effects	167	36.5%	(32.1–41.1)	33.3%	(29.2–37.6)
Negative interaction with provider	32	7.0%	(4.8–9.7)	6.4%	(4.4–8.9)
Positive interaction with provider	2	0.4%	(0.1–1.6)	0.4%	(0.1–1.4)
Instructional	20	4.4%	(2.7–6.7)	4.0%	(2.5–6.1)
Other	14	3.1%	(1.7–5.1)	2.8%	(1.5–4.6)
Structural/Access Barriers	14	3.1%	(1.7–5.1)	2.8%	(1.5–4.6)
Reproductive/Clinical Considerations	19	4.1%	(2.5–6.4)	3.8%	(2.3–5.9)

Percentages are presented using different denominators: “percentage of videos” reflects the proportion of total videos (n = 458) in which the topic appears, while “percentage of total topic mentions” reflects the proportion of all topic occurrences across videos (n = 502).

**Table 5:** Inter-rater reliability (ICC, two-way random, absolute agreement) for Modified DISCERN and GQS scores.

Measure	ICC (Single Measures)	95% CI	Interpretation
Modified DISCERN	0.698	(0.607–0.765)	Moderate
GQS	0.771	(0.730–0.806)	Good

Inter-rater reliability was assessed using the intraclass correlation coefficient (ICC) based on a two-way random-effects model with absolute agreement (single measures). ICC values were interpreted as follows: <0.50 = poor, 0.50–0.75 = moderate, 0.75–0.90 = good, and >0.90 = excellent agreement. Confidence intervals represent 95% confidence limits.

**Table 6:** Comparison of user engagement metrics by video type and source.

Variable	Category	Likes (Mean ± SD)	*p*	Views (Mean ± SD)	*p*	Comments (Mean ± SD)
Video type	Educational	48,301 ± 196,701	0.409	1,663,989 ± 7,459,098	0.069	698 ± 2722
	Non-educational	67,701 ± 271,122	—	784,721 ± 2,440,949	—	899 ± 2923
Video source	Individual	57,859 ± 239,644	0.498	1,046,923 ± 5,033,588	0.255	806 ± 2797
	Organizational	86,354 ± 300,456	—	2,031,276 ± 5,055,058	—	987 ± 3390
Creator qualification	Physician	35,524 ± 91,243	0.251	949,304 ± 2,500,767	0.011	813 ± 2681
	Non-physician HCP	99,484 ± 372,336	—	2,856,462 ± 11,900,819	—	1064 ± 4099
	Other	60,529 ± 248,008	—	799,007 ± 2,397,542	—	768 ± 2553

Values are presented as mean ± standard deviation. *p* values were derived from independent-sample *t*-tests (video type and source) and one-way ANOVA (creator qualification). Bolded *p*-values indicate statistical significance at *p* < 0.05. HCP = healthcare provider. Omnibus significance was observed for views by creator qualification; however, Games–Howell post hoc testing indicated no single pairwise difference remained statistically significant after adjustment for unequal variances and multiple comparisons.

**Table 7:** Comparison of informational quality and false claims by creator qualification.

Variable	Categories	Physician	Non-Physician Healthcare Providers	Others	*p* Value
False Claims	Yes	5 (4.6%)	7 (10.8%)	32 (11.2%)	0.132
	No				
Average Modified DISCERN Score	-	2.9 ± 0.6	2.5 ± 0.8	1.3 ± 0.6	<0.001
Average GQS Score	-	2.3 ± 0.5	2.2 ± 0.7	1.9 ± 0.5	<0.001

**Table 8:** Post hoc Games–Howell pairwise comparisons of informational quality scores by creator qualification.

Variable	Group	Mean Difference	*p*-Value	95% CI (LCL–UCL)
Average Modified DISCERN Score				
Physician	Non-physician healthcare providers	0.3056	0.016	0.046–0.565
	Others	1.5708	<0.001	1.420–1.721
Non-physician healthcare providers	Others	1.2652	<0.001	1.026–1.505
Average GQS Score				
Physician	Non-physician healthcare providers	0.0177	0.981	−0.210–0.246
	Others	0.3814	<0.001	0.243–0.520
Non-physician healthcare providers	Others	0.3637	<0.001	0.160–0.567

Pairwise comparisons in Modified DISCERN and Global Quality Scale (GQS) scores were assessed using Games–Howell post hoc tests following significant omnibus ANOVA results. The Games–Howell method accounts for unequal variances and unequal group sizes and is robust to violations of normality in large samples. Mean differences are reported for interpretability within the context of parametric post hoc comparisons. Positive mean differences indicate higher scores in the first-listed group.

## Data Availability

The data supporting the findings of this study were derived from publicly available TikTok videos. Due to platform terms of service, potential changes in content availability over time, and ethical considerations related to user privacy, the full dataset, including video URLs, cannot be publicly shared. However, aggregated data and the list of video URLs are available from the corresponding author upon reasonable request.
